# The Role of 4D Flow MRI-derived Wall Shear Stress in Aortic Disease: A Comprehensive Review

**DOI:** 10.31083/RCM26735

**Published:** 2025-03-05

**Authors:** Ying Liu, Xiaolin Mu, Yixin Wang, Zhe Xu, Yang Song

**Affiliations:** ^1^Department of Radiology, Central Hospital of Dalian University of Technology, 116033 Dalian, Liaoning, China; ^2^Department of Graduate School, Dalian Medical University, 116044 Dalian, Liaoning, China

**Keywords:** 4D flow, magnetic resonance imaging, wall shear stress, aortic

## Abstract

Aortic diseases, such as aortic dissection and aortic rupture, often lead to catastrophic complications, significantly increasing morbidity and mortality. Population-based screening for early detection in asymptomatic individuals is not feasible due to high costs and practical challenges. However, recent advancements in four dimensions (4D) Flow magnetic resonance imaging (MRI) offer a comprehensive tool for evaluating hemodynamic changes within the aortic lumen. This technology allows for the quantification and visualization of flow patterns and the calculation of advanced hemodynamic parameters, such as wall shear stress (WSS). WSS is crucial in the development, risk stratification, and surgical outcomes of aortic diseases and their complications, enabling noninvasive and quantitative screening of high-risk populations. This review explores the current status and limitations of 4D flow MRI-derived WSS imaging for aortic disease.

## 1. Introduction

Complications from aortic disease, such as aortic dissection and aortic rupture, are often catastrophic, resulting in increased morbidity and mortality [1]. The development of aortic disease is closely related to the luminal hemodynamic environment, with wall shear stress (WSS) playing an important role. Under physiological conditions, vascular endothelial cells possess mechanosensors that detect the magnitude and direction of WSS, including ion channels, cell-cell junctions, G protein-coupled receptors, integrins, and glycocalyx [2, 3, 4]. These mechanosensors regulate the expression of relevant genes and their proteins by activating signal transduction pathways [5]. Normal WSS maintains vascular homeostasis and exerts anti-proliferative, anti-apoptotic, anti-inflammatory and anti-thrombotic effects. Conversely, abnormal WSS can lead to vascular dysfunction, inflammation and thrombosis [6, 7]. This underscores the importance of WSS in the physiological stages and progression of vascular diseases.

The aorta is mainly evaluated by Color Doppler ultrasound, computed tomography angiography (CTA), digital subtraction angiography (DSA) and other techniques. But these techniques have some defects, for example, ultrasound is susceptible to gas and the quality and accuracy of its images are related to the level of the imaging physician; CTA and DSA require the introduction of contrast agents, which can only obtain information on the morphology of the aorta. They need to be combined with hydrodynamic post-processing software in order to provide hemodynamics. Computational fluid dynamics (CFD) is a common hemodynamic measurement that provides high spatial and temporal resolution [8], but it simulates the real blood flow information by computer modeling, and the data obtained are to some extent virtual. Four dimensions (4D) flow magnetic resonance imaging (MRI) is a new non-invasive, contrast-free imaging technique that can truly reflect the status of intravascular blood flow and retrospectively analyze the aortic hemodynamic information to dynamically evaluate the abnormal blood flow status in the aorta for early intervention. This article summarizes the current status and limitations of 4D flow MRI-derived WSS in aortic disease, aiming to provide new insights into the clinical management of aortic disease and its complications.

## 2. 4D Flow MRI-derived WSS Imaging Technology and Post-processing Software

Currently, Siemens, Philips and GE MRI vendors are able to perform 4D Flow MRI acquisitions, and the WSS obtained from two consecutive 4D Flow scans on the same magnetic resonance (MR) scanner has good consistency and reproducibility [9, 10, 11, 12]. However, the quantitative values of WSS obtained by different MR vendors have not been standardized [13]. In addition, there was variability in WSS values between 1.5 T and 3.0 T from the same MR supplier [14, 15].

**Fig. 1.**
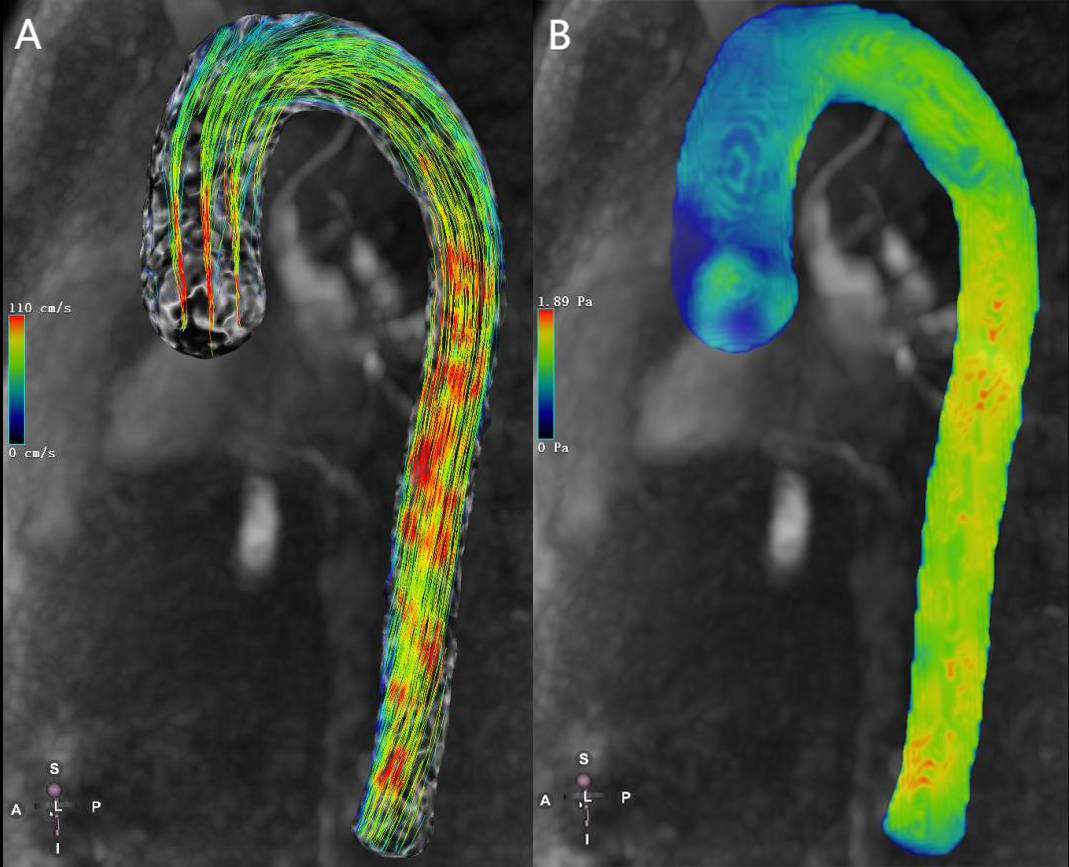
**4D flow magnetic resonance imaging (MRI)–based visualization of aortic hemodynamics in a healthy volunteer**. (A) 4D-flow-Image streamlines for the aorta. (B) 4D-flow-Image wall shear stress for the aorta. The color gradient change reflects the magnitude of velocity and wall shear stress (WSS). Red and blue are the maximum and minimum values, respectively. 4D, four dimensions.

Efficient post-processing software is essential for the quantification and visualization of hemodynamic parameters in both scientific research and clinical applications. The example of 4D flow MRI–based visualization of aortic hemodynamics is shown in Fig. 1. Current post-processing software includes CAAS 5.1 (Pie Medical Imaging, Maastricht, Limburg, the Netherlands), CVI42 6.0.2 (Circle Cardiovascular Imaging, Calgary, Alberta, Canada), GT Flow 3.1.14 (GyroTools, Zurich, Switzerland), iT Flow 1.9 (Cardio Flow Design Inc., Chiyoda-ku, Tokyo, Japan) and MEVISFlow 10.3 (Fraunhofer MEVIS, Bremen, Germany). In addition, some researchers have developed advanced flow analysis parameters using MATLAB R2022b (MathWorks Inc., Natick, MA, USA) or other programming languages to visualize the advanced flow data using post-processing tools in the field of fluid dynamics, such as Ensight and Paraview. It is important to note that there are significant differences in WSS quantitative reference values derived from different post-processing software [16]. These discrepancies may be related to variations in background phase offset corrections, contour splitting, and software algorithms.

Limitations: The lack of uniformity in WSS measurement limits its clinical application. However, it has been suggested that aortic remodeling can be predicted by differentiating between areas of high and low WSS [17]. In the future, standardization of scanning protocols and uniformity of post-processing methods are needed before 4D Flow can be introduced into routine clinical applications.

## 3. 4D Flow MRI-derived WSS Clinical Applications

In recent years, 4D Flow MRI-derived WSS is a hemodynamic parameter highly relevant to aortic disease. It plays a significant role in the occurrence, development, risk stratification, and surgical evaluation of aortic diseases such as aortic aneurysm, aortic dissection, aortic atherosclerosis, bicuspid aortic valve, and Marfan syndrome (Table 1, Ref. [12, 18, 19, 20, 21, 22, 23, 24, 25, 26, 27, 28, 29, 30, 31, 32, 33, 34, 35, 36, 37, 38, 39, 40, 41, 42, 43, 44, 45, 46, 47, 48, 49, 50, 51, 52, 53, 54, 55, 56, 57, 58, 59]).

**Table 1.** **Research status of the application of 4D Flow MRI-derived WSS in aortic diseases**.

Type of disease	Author	Date published	Research	Research Finding
Aneurysm	Natsume *et al*. [26]	2017	16 aortic arch aneurysms, 8 young healthy volunteers and age-matched control subjects	Aneurysm geometry affects WSS distribution
	Jamaleddin Mousavi *et al*. [21]	2021	2 ATAA, 2 healthy subjects	Computational modeling coupling hemodynamics with mechanobiology as a promising approach for exploring aneurysm progression
	Ramaekers *et al*. [20]	2021	25 TAA, 22 controls	Asymmetrically distributed peak WSS values in TAA
	Salmasi *et al*. [24]	2021	10 ATAA	Elevated WSS values associated with aortic wall degradation in ATAA disease
	Trenti *et al*. [18]	2022	18 AAA, 22 age-matched controls, 23 young subjects	RRT as a marker for abnormal AAA hemodynamics
	Aalbregt *et al*. [12]	2024	22 asymptomatic AAA	A 4D flow MRI is robust for assessing the hemodynamics within AAAs
	Bouaou *et al*. [19]	2024	17 ATAA, 17 healthy controls, 13 younger healthy subjects	Flow and pressure indices associated with WSS
	Zeng *et al*. [25]	2024	11 TAAA, 19 AAA, 21 controls	Lower WSS in TAAA
Dissection	Veger *et al*. [28]	2021	A porcine aorta dissection model	Strict heart rate control is of major importance in reducing the mean and peak WSS in uncomplicated acute TBAD
	Ruiz-Muñoz *et al*. [27]	2022	54 Patients with chronic AD	WSS positively correlated with aortic growth rate
	Stokes *et al*. [29]	2023	A 56-year-old male patient with chronic TBAD	Oscillatory shear is highly sensitive to inlet velocity distribution and flow volume
Atherosclerosis	Harloff *et al*. [30]	2010	62 aortic atherosclerosis, 31 healthy volunteers	4D flow MRI was successfully used to analyze multiplanar WSS distribution
	Markl *et al*. [33]	2013	70 patients with complex plaques, 12 healthy volunteers	Decreased WSS and increased OSI in patients with atherosclerosis
	Winter *et al*. [31]	2021	5 wildtype and 5 *ApoE* mice	A new post processing method with 4D flow MRI
	Andelovic *et al*. [32]	2021	6 healthy wildtype and 6 atherosclerotic *ApoE* mice	WSS_circ_ as potential marker of plaque size and composition in advanced atherosclerosis
Bicuspid aortic valve	Barker *et al*. [43]	2012	15 BAV, 45 controls	Increased and asymmetric WSS according to BAV fusion pattern
	Meierhofer *et al*. [36]	2013	18 BAV, 18 healthy individuals	Increased WSS in healthy BAV individuals
	van Ooij *et al*. [35]	2015	13 BAV, 10 controls	Elevated WSS correlated with peak systolic velocity
	Guzzardi *et al*. [22]	2015	20 BAV	Regions of increased WSS show greater medial elastin degradation
	Shan *et al*. [41]	2017	50 BAV, 15 TAV	Severe aortic insufficiency or stenosis resulted in further elevated WSS
	van Ooij *et al*. [45]	2017	270 BAV, 245 TAV with aortic dilatation, 56 healthy subjects	Increased WSS in BAV and TAV with AS
	Rodríguez-Palomares *et al*. [44]	2018	101 BAV, 20 healthy subjects	Different BAV-phenotypes present different flow patterns
	Farag *et al*. [40]	2018	48 BAV, 25 healthy individuals	Increased WSS in BAV patients with AS
	Bollache *et al*. [37]	2018	27 BAV	Increased aortic valve-mediated WSS associated with elastic fiber thinning
	Soulat *et al*. [38]	2022	72 BAV, 136 controls	WSS correlated with AAO dilation
	Guala *et al*. [23]	2022	47 BAV	Circumferential WSS predict progressive dilation of the ascending aorta in patients with BAV
	Minderhoud *et al*. [39]	2022	32 BAV, 28 healthy controls	WSS angle associated with aortic growth
	Trenti *et al*. [42]	2024	42 BAV, 22 normal controls	Elevated OSI in BAV with aortic regurgitation
	Maroun *et al*. [34]	2024	20 BAV, 125 controls	The long-term stability of 4D flow MRI-derived WSS and WSS-derived heatmaps
Aortic valve replacement	von Knobelsdorff-Brenkenhoff *et al*. [49]	2014	38 AVR, 9 healthy controls	AVR types affect WSS
	Trauzeddel *et al*. [53]	2016	17 TAVI, 12 AVR, 9 healthy controls	Increased WSS after TAVI and AVR compared to healthy controls
	van Kesteren *et al*. [50]	2018	14 stented and 14 stentless bioprosthesis	Lower WSS for stentless prosthesis
	Bissell *et al*. [51]	2018	30 AVR, 30 patients with a native aortic valve, 30 healthy subjects.	Decreased WSS after mechanical AVR or Ross procedures
	Bollache *et al*. [47]	2018	33 patients received operation, 20 control patients did not	Proximal aortic WSS decreased after AVR
	Farag *et al*. [52]	2019	14 post-TAVR patients and 10 healthy controls	Increased WSS in the ascending aorta after TAVR
	Komoriyama *et al*. [48]	2021	32 Pre-TAVR and post-TAVR patients	TAVR improves blood flow dynamics
	Wiesemann *et al*. [46]	2023	7 patients received an AVR, 13 control patients did not	Decreased WSS compared to patients who were not operated on
Marfan syndrome	Geiger *et al*. [54]	2013	24 MFS, 12 older healthy volunteers	Higher WSS at the inner curvature in the proximal AAO and at the anterior part in the more distal AAO
	Wang *et al*. [57]	2016	20 MFS and 12 age-matched normal subjects	Lower WSS_axial_ in the aortic root and the WSS_circ_ in the arch
	van der Palen *et al*. [58]	2017	25 MFS, 21 healthy controls	Lower WSS in pediatric MFS patients
	Geiger *et al*. [55]	2017	19 adolescent MFS, 10 healthy volunteers	Lower WSS in the inner proximal DAO in a 3-year follow-up
	Guala *et al*. [59]	2019	75 Marfan, 48 healthy subjects	Abnormal circumferential WSS in Marfan patients
	van Andel *et al*. [56]	2022	55 MFS, 25 healthy subjects	Deviant directed WSS in the DAO was more frequently seen in male patients and in patients with a HI mutation type

ATAA, ascending thoracic aortic aneurysm; TAA, thoracic aortic aneurysm; AAA, abdominal aortic aneurysm; RRT, relative residence time; TAAA, thoracoabdominal aortic aneurysm; TBAD, type B aortic dissection; AD, aortic dissection; OSI, oscillatory shear stress; ApoE, apolipoprotein E-deficient; WSS_circ_, circumferential WSS; BAV, bicuspid aortic valve; TAV, tricuspid aortic valve; AS, aortic valve stenosis; AAO, ascending aorta; AVR, aortic valve replacement; TAVR, transcatheter aortic valve replacement; MFS, Marfan syndrome; WSS_axial_, axial WSS; DAO, descending aorta; HI, haploinsufficient.

Additionally, some scholars have derived the oscillatory shear index (OSI) based on WSS [60, 61], which indicates the degree of directional change of WSS during a cardiac cycle [62, 63]. A high OSI implies significant directional changes in WSS, reflecting large shear stress fluctuation. Another study introduced relative residence time (RRT), a parameter based on the values of WSS and OSI [18]. RRT can identify areas with low WSS and high OSI, which are prone to aortic diseases such as plaque formation. Therefore, high RRT can be used to locate high-risk areas [18, 64].

### 3.1 Aortic Aneurysm

Risk stratification of aortic aneurysms is a prominent and challenging research topic, with increasing attention being paid to the influence of hemodynamics on aneurysms [19, 20, 21]. WSS is closely related to aneurysm formation, growth, and rupture, and it changes continuously during aneurysm progression.

A series of histopathological studies have revealed that high WSS leads to dysregulation of the extracellular matrix and degeneration of elastic fibers, resulting in thinning of the arterial wall, promoting aneurysm formation [22, 65, 66]. A study utilizing 4D Flow MRI has reached similar conclusions, showing that elevated WSS is strongly associated with an increased rate of aortic diameter growth. In particular, high circumferential WSS may be an independent predictor of aneurysm formation [23].

**Fig. 2.**
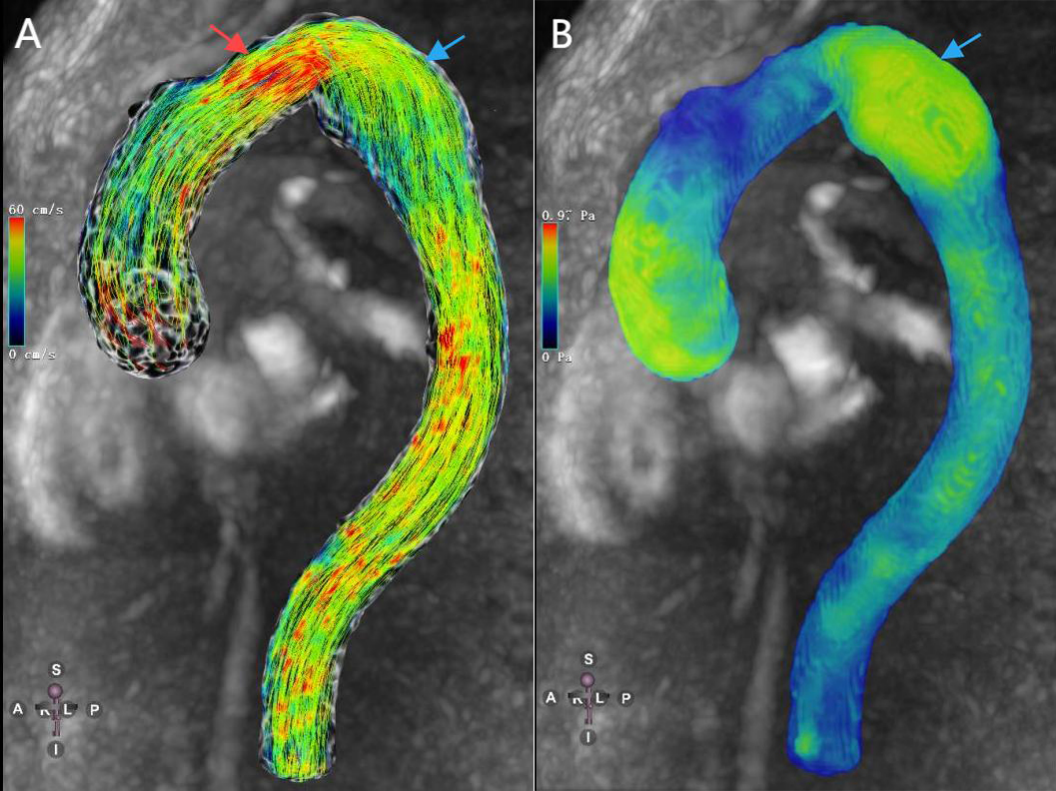
**A patient with descending aortic aneurysm**. (A) 4D-flow-Image with streamlines demonstrates low velocity inside the thoracic aortic aneurysm (blue arrow) and high velocity around the aneurysm (red arrow). (B) 4D-flow-Image demonstrates high WSS inside the thoracic aortic aneurysm (blue arrow).

During aneurysm evolution, there are morphological and hemodynamic differences between aneurysms with different WSS, and both high and low WSS potentially contributing to aneurysm growth and rupture [67, 68]. Salmasi *et al*. [24] assessed the relationship between preoperative 4D Flow MRI images and postoperative tissue specimen characteristics in patients with ascending aortic aneurysms, finding that areas of high WSS were associated with aortic wall thinning, elastin abundance, and decreased smooth muscle cell counts. This suggests that degradation and thinning of the aneurysm wall are associated with hemodynamic impairment and high WSS (Fig. 2). Conversely, low WSS leads to inflammatory cell-mediated endothelial injury and apoptosis, with low and oscillating WSS areas being prone to plaque formation. This results in large, thick-walled aneurysms due to the combination of the inflammatory response and plaque buildup [69].

Aortic aneurysms often exhibit vortex or helical flow, with lesion areas showing low WSS and high OSI [25, 26, 60, 61]. These abnormal hemodynamics tend to promote aortic atherosclerosis, which in turn leads to progressive aortic dilatation and increases the risk of aneurysm rupture. Additionally, it has been found that RRT seems to be a more powerful predictor of hemodynamic changes in aortic aneurysms than the commonly used OSI [18], as it accounts for both the magnitude and direction of WSS.

### 3.2 Aortic Dissection

**Fig. 3.**
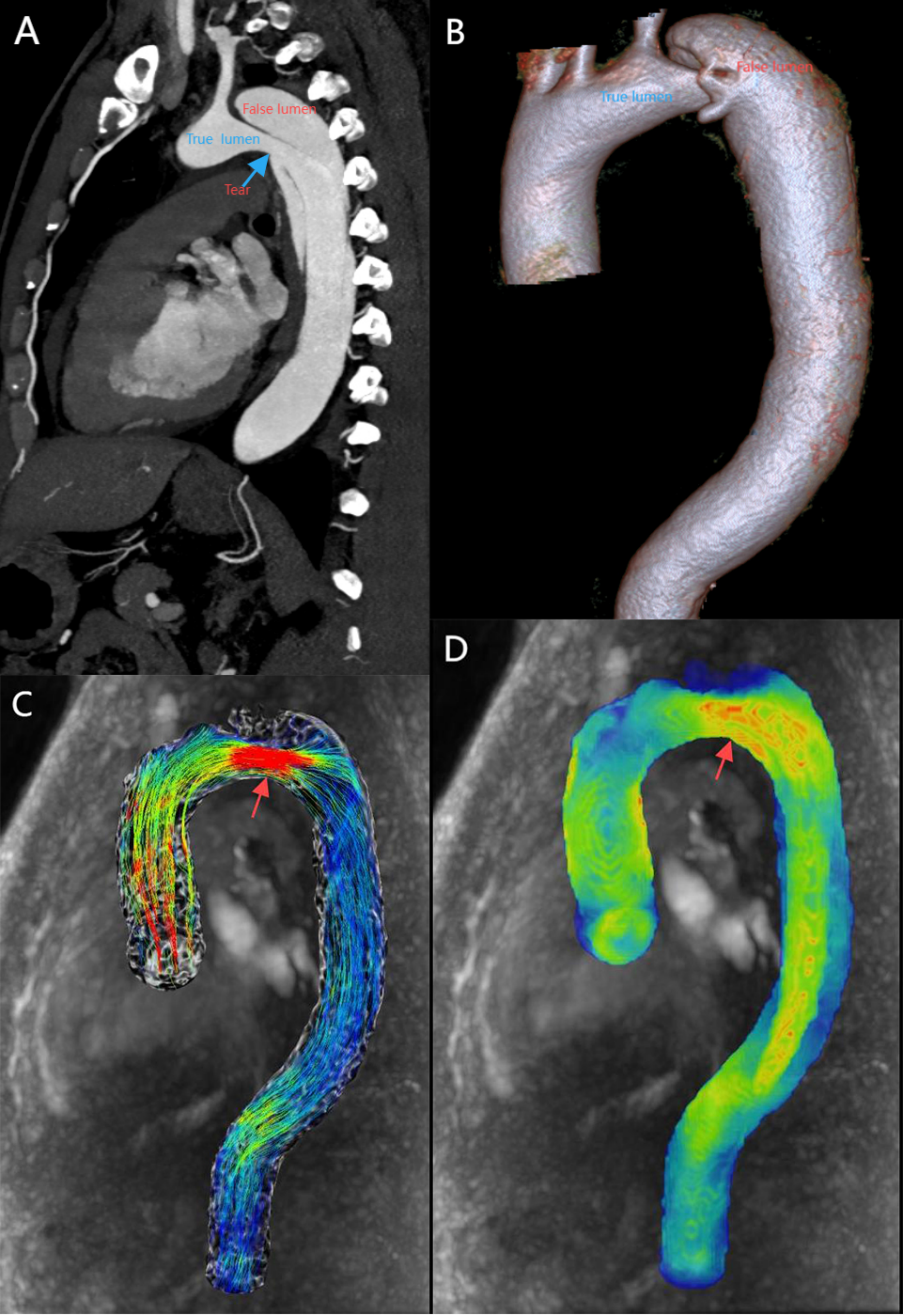
**A patient with stanford type B aortic dissection**. (A) 2D sagittal view, showing true and false lumen as well as entry tear (blue arrow). (B) 3D VR displays true lumen, false lumen. (C) 4D-flow-Image with streamlines demonstrates high velocity at the entry tear (red arrow). (D) 4D-flow-Image demonstrates high WSS at the entry tear (red arrow). 2D, two dimensions; 3D, three dimensions; VR, volume rendering.

Aortic dissection is one of the common types of acute aortic syndrome (AAS) with rapid onset and high mortality. Early screening for the potential risk of aortic dissection in high-risk groups would facilitate clinical preventive measures and reduce patient mortality. Fig. 3 shows aortic dissection evaluated by imaging modalities. Research has found that the area of highest WSS is highly coincident with the location of the tear in stanford type A aortic dissection [70]. This suggests that increased WSS may be an important factor in endovascular injury. Patients with stanford type B aortic dissection can be treated medically or surgically based on clinical assessment, and real-time monitoring of the dynamic evolution of the aortic dissection is mandatory. Increased aortic diameter and partial thrombosis of the false lumen are associated with late adverse events in type B aortic dissection [71, 72]. WSS may be a good indicator for monitoring these changes. On the one hand, WSS is positively correlated with the aortic growth rate [27], which could be used as a predictor of the distant expansion of aortic dissection. On the other hand, low WSS, high RRT, flow patterns are correlated with thrombosis [73]. There is increasing evidence to indicate that 4D flow MRI-based hemodynamics plays an important role in aortic dissection management and prognosis [27, 28, 29, 74].

### 3.3 Aortic Atherosclerosis

WSS is one of the most important hemodynamic parameters in the formation and progression of atherosclerosis. Previous studies have demonstrated that persistent abnormal WSS can cause pathophysiological changes such as vascular remodeling, cell death, extracellular matrix degradation, and pro-inflammatory responses, which can promote plaque formation [75, 76, 77]. 4D Flow MRI provides a powerful tool for monitoring the distribution of aortic WSS in patients with atherosclerosis, allowing measurement of vessel wall parameters in the region of interest from any direction and angle [30, 31]. Circumferential WSS may be an important parameter in the noninvasive assessment of atherosclerotic plaque characteristics, correlating with plaque size, macrophage content, calcification, and necrotic core area [32], suggesting that abnormal circumferential WSS is critical for plaque growth and progression towards vulnerability. A study [33] analyzing 140 complex plaque locations using 4D Flow MRI found that aortic branches, bifurcations, or bends near the aorta are susceptible to disturbed and flocculated flow, generating areas of low and oscillating WSS, which are common areas of plaque formation. 4D Flow MRI-based study confirms that low WSS and high OSI promote the occurrence and development of atherosclerotic plaque [78, 79].

The rupture of atherosclerotic plaques can lead to vascular obstruction and trigger serious adverse consequences. High WSS increases metalloproteinase activity, accelerates angiogenesis and transformation, thereby enhancing plaque vulnerability and inducing plaque rupture [80, 81]. There are few studies applying 4D Flow to predict aortic plaque rupture. In a study of carotid plaques, high-risk plaques were found to have higher WSS, which proved that high WSS might be related to plaque rupture and more likely to cause cerebrovascular events [82].

### 3.4 Bicuspid Aortic Valve 

The bicuspid aortic valve (BAV) is a common malformation of the aortic valve, occurring most often in males. 4D Flow MRI-derived WSS has demonstrated great potential for hemodynamic measurements in patients with BAV [34, 35]. Even in patients with normal BAV, the ascending aorta shows elevated WSS [36]. Areas with increased WSS are prone to aortic extracellular matrix dysregulation and elastic fiber thinning, which are associated with subsequent aortic disease [22, 37]. A prospective longitudinal study [23] found that elevated circumferential WSS predicted the growth rate of ascending aortic diameter in patients with BAV and may be a marker for the risk of ascending aortic dilatation. Other studies have confirmed the relationship between high WSS and aortic growth [38, 39]. BAV can be associated with valvular dysfunction, with elevated WSS observed in cases of aortic stenosis [40, 41] and elevated OSI observed in cases of aortic regurgitation [42].

Hemodynamic changes are also associated with the BAV valve fusion phenotype [43]. Right-left bicuspid aortic valve (RL-BAV) patients present a higher axial WSS at the aortic root while right-non coronary bicuspid aortic valve (RN-BAV) present a higher circumferential WSS in the mid and distal ascending aorta (AAO) [44]. However, moderate-to-severe aortic stenosis blurs the difference in WSS between these valve types, as the presence of aortic stenosis dominates ascending aortic hemodynamics irrespective of the valve fusion phenotype [41, 45].

### 3.5 Aortic Valve Replacement

Aortic valve replacement (AVR) significantly improves symptoms, quality of life, and prolongs survival in patients with aortic stenosis. A 4D Flow MRI-based study found improved aortic hemodynamics and reduced WSS after AVR compared with preoperative patients with aortic disease [46, 47, 48]. Different types of AVR present different hemodynamic changes post-surgery [49, 50]. Bissell *et al*. [51] compared the mechanical parameters of blood flow in 30 patients with BAV who underwent AVR using different surgical modalities and found that the aorta returned to a normal flow pattern in patients after mechanical valve replacement or the Ross procedure. However, higher WSS and abnormal spiral flow persisted in patients after bioprosthetic AVR. Transcatheter aortic valve replacement (TAVR) is an effective alternative to surgical aortic valve replacement (SAVR) for elderly and high-risk patients with aortic stenosis [83]. Ascending aortic WSS was increased and asymmetrically distributed after TAVR compared to healthy controls [52, 53].

### 3.6 Marfan Syndrome

Marfan syndrome (MFS) is a hereditary connective tissue disorder in which aortic complications are the main cause of death, including aortic dissection and aortic aneurysm rupture. Early prediction of the development of aortic complications in patients with Marfan syndrome by hemodynamic parameters is of great significance. Local helix flow patterns in the ascending aorta and proximal descending aorta were confirmed in a study of adolescent MFS patients using 4D Flow MRI [84]. This resulted in a heterogeneous regional WSS distribution, with elevated WSS in the proximal inner curvature of the ascending aorta [54] and decreased segmental WSS in the inner proximal descending aorta [55]. At follow-up after 3 years, WSS was reduced in the inner proximal descending aorta, which may be related to the risk of aortic dissection [55, 56]. Some scholars have disagreed, suggesting that proximal WSS in the ascending aorta is reduced, hypothesizing that the inconsistent results are due to the fact that young MFS patients exhibit higher WSS [57, 58]. In addition, both circumferential and axial WSS of the proximal descending aorta are independently correlated with local lumen diameter, and decreased circumferential WSS may be one of the early markers of descending aortic dilatation in patients with Marfan syndrome [59]. Patients with MFS need to be monitored for a long period of time, and in the future, 4D Flow MRI could be added as part of regular observations, which may help clinicians to predict the occurrence of MFS complications at an early stage.

## 4. Shortcomings and Prospects

Currently, 4D flow MRI technology still has many shortcomings and is mainly used for small-sample studies. First of all, 4D flow MRI scanning time is long, but the clinical environment still needs shorter imaging time, and image resolution is relatively low, which makes it difficult to accurately recognize aortic complex anatomies and cases, and at the same time affects the accuracy of WSS parameter values. In the future, we need to improve the K-space sampling, compression perception technology and deep learning to shorten the scanning time and enhance the spatial and temporal resolution. Second, the variability in parameters obtained from different post-processing vendors makes it difficult to establish a unified normal reference value for WSS. Therefore, developing and establishing a standardized workflow is crucial. Third, research based on 4D Flow MRI-derived WSS in aortic disease is limited. There is a need for more and larger prospective cohorts to explore how this technology could be integrated into existing clinical workflows and its expected impact on patient management and treatment outcomes.

## 5. Conclusions 

In conclusion, 4D flow MRI-derived WSS is a promising tool for assessing the occurrence and development, risk stratification, and surgical efficacy of aortic disease and its complications.
